# New understandings meet old treatments: putting a contemporary face on established protocols

**DOI:** 10.1186/s40337-024-00983-4

**Published:** 2024-02-09

**Authors:** Susan M. Byrne, Anthea Fursland

**Affiliations:** 1 University of Western Australia, Perth, WA Australia; 2 The Swan Centre, Perth, WA Australia

**Keywords:** Eating disorders, Evidence-based treatment, Adapting treatments, Expanding research design

## Abstract

In the twenty years since the publication of the most widely used treatment manuals describing evidence-based therapies for eating disorders, there have been some substantial advances in the field. New methods of delivering treatments have been trialled and our perception of mental health has advanced; significant cultural changes have led to shifts in our societal landscape; and new technologies have allowed for more in-depth research to be conducted. As a result, our understanding of eating disorders and their treatment has broadened considerably. However, these new insights have not necessarily been translated into improved clinical practice. This paper highlights the changes we consider to have had the greatest impact on our work as experienced clinical psychologists in the field and suggests a list of new learnings that might be incorporated into clinical practice and research design.

## Abstract (plain language summary)

In the field of eating disorders the most commonly used manualised treatments are nearly twenty years old. There has been much progress in the field since then in terms of technologies, understandings and social changes. In this paper, two experienced clinical psychologists describe some of the more recent developments in the field and highlight ways to incorporate the new learnings into clinical practice and research design.

## Introduction

In our long careers (over thirty years) in the eating disorders (EDs) field we have seen many changes. At the turn of the century, Cognitive Behaviour Therapy for Bulimia Nervosa (CBT-BN) was being developed into a transdiagnostic treatment by Professor Christopher Fairburn and his colleagues at Oxford University. However, it was only a few years before the publication of *Cognitive Behaviour Therapy and Eating Disorders* in 2009 [1] that Enhanced Cognitive Behaviour Therapy (CBT-E) began to be disseminated. Likewise, while family therapy for anorexia nervosa (AN) was being refined by Ivan Eisler and colleagues at the Maudsley Hospital in the nineties, it was only when the *Treatment Manual for Anorexia Nervosa: A Family Based Approach* [2] was published in 2001, with related research indicating positive outcomes, that clinicians became knowledgeable about, and trained in, Family Based Treatment (FBT). While these two therapies continue to be the leading evidence-based treatments (EBTs) for EDs, treatment outcomes remain limited.

In this paper we share our clinical experience of the past three decades to illustrate how the ED field has changed as more knowledge has emerged. We discuss the two most broadly used EBTs, CBT-E and FBT, in terms of their relevance today, their limitations, and how they might be expanded to encompass these changes. We stand by our support of these two EBTs and other EBTs that have been developed over the past two decades, e.g., The Maudsley Anorexia Nervosa Treatment for Adults (MANTRA) [3] and Specialist Supportive Clinical Management (SSCM) [4], while examining their significance in the current climate.

Some of the changes that have had the greatest impact on our work over the past two decades have stemmed from new research into, for example, the impact of starvation on the brain, the genetic and neurobiological underpinnings of EDs, and the role of the microbiome. Other changes reflect broader sociocultural shifts such as greater acknowledgement of diversity including neurodiversity and diversity in terms of gender, ethnicity and body size/shape; greater acceptance of the value of lived experience in both research design and treatment, as well as the advent of social media and recognition of problems related to food insecurity. Additional changes have followed on from revisions in the DSM and from significant advances in treatments for other psychiatric conditions. We make some suggestions about how we might build on treatment protocols that are now two decades old and how we might adapt our approach to ED research in light of this new information.

## Personalizing treatment

Whilst continuing to ground our work with patients in EBTs, we propose moving toward more individualised, flexible approaches than those prescribed in treatment manuals created over twenty years ago. Personalising treatment is an overarching recommendation that sits above all our suggestions, presented below, about ways of updating current EBTs.

In our view, personalising treatment involves developing competence to make treatments more appropriate by: (1) providing truly relevant, up-to-date psychoeducation, based on recent research findings, that is specific to the individual’s presentation; (2) making decisions about the treatment delivery that is most suited to the patient; (3) collaboratively developing treatment goals that reflect the patient’s current needs and values; (4) adapting or nuancing treatments to meet the needs of diverse populations; (5) paying attention to the sociocultural shifts that have occurred over the past twenty years; (6) modularizing EBTs, using adjunctive evidence-based techniques developed for other conditions; and (7) updating our research designs in order to address the questions we most need to answer in the most economical way. At present, there is little evidence that making personalised adaptations to EBTs leads to improved outcomes for people with eating disorders. We believe that this concept should be a priority for future research and we hope that the suggested new topics for consideration that we list below may spark new research.

### #1: Psychoeducation using 21st Century research findings

Psychoeducation is a crucial component of CBT-E and FBT but recent research has provided us with more detailed information about the psychoeducation topics that are mentioned in these treatment manuals. With a greater appreciation of the effects of starvation on the brain, our increased knowledge of genetics, neurobiology, neuroplasticity and the gut-brain axis, we emphasize the importance of offering comprehensive psychoeducation early, starting during the assessment phase, to help patients and their families understand the illness and empower them to make informed decisions about their treatment and the recovery process. We suggest that clinicians update their knowledge about these key points.

#### The effects of starvation: beyond the Minnesota starvation experiment

While we have always known about the Keys Semi-Starvation Study [5], there is more appreciation nowadays of the far-reaching effects of starvation on the body and the brain, with Magnetic Resonance Imaging studies highlighting the significant neurological changes that follow prolonged malnutrition [6]. Additionally, there is recognition that the effects of starvation, including the resulting medical instability, can be present in individuals at any size [7].

#### Genetics, neurobiology and neuroplasticity

An explosion of research over the last decade has led to a greater understanding of the genetic, epigenetic and neurobiological underpinnings of EDs particularly in regard to Anorexia Nervosa (AN) [8, 9], Avoidant Restrictive Feeding and Intake Disorder (ARFID) [10], and Binge Eating Disorder (BED) [11]. Genome Wide Association Studies have accrued large enough samples to confirm evidence from twin and family studies of a strong genetic component to EDs [8, 12, 13]; they are highly heritable illnesses, not a “lifestyle choice.” These studies have also highlighted significant genetic correlations with other psychiatric conditions, including obsessive compulsive disorder, depression and anxiety [8] and AN-associated genes have been found to not only impact the brain, but also the gut and metabolic factors leading researchers to propose a reconceptualization of AN as a “metabo-psychiatric” disorder [14–16]. Neuroimaging evidence has confirmed the notion of a genetically driven, neurobiologically based AN temperament that influences vulnerability to and persistence of the illness [6, 17]. The role of epigenetics in the expression of EDs has also become better understood: we have learned about the complex links between genes and nutritional and environmental factors (e.g., calorie deficit, stress, trauma) that can modify gene expression and/or “switch on” genes related to the development and maintenance of EDs [17, 18]. Finally, recent neurobiological research has encompassed the notion of neuroplasticity and neuromodulation which increases hope for the development of more successful therapies [19–21].

#### The influence of the microbiome

Information about the gut-brain axis has led to a recognition of the influential role of the gut microbiome on mental health in general [22] and EDs in particular [23–26]. Gastrointestinal symptoms and problems such as irritable bowel syndrome, post-prandial fullness, reflux, constipation, bloating and abdominal pain are prevalent in individuals with EDs and are associated with high levels of distress and impairment [7, 27, 28]. There is an increasing body of research focusing on the complex links between the gut microbiome, gastrointestinal disturbances and EDs across the whole spectrum of eating pathology.

### #2: Redefining treatment delivery in the 21st Century

#### The treatment team

The original treatment manuals for both CBT-E and FBT promote treatment by a *single* therapist, usually a mental health professional in isolation (with FBT requiring regular appointments with a medical practitioner). However, with the recognition of EDs as complex, genetically based neuropsychological disorders we have moved beyond the idea of treatment being delivered by a single therapist. It is generally accepted that good treatment for EDs requires a multi-disciplinary team with a range of knowledge and skills [29, 30]. We have come to value the special contribution of dietitians [31, 32] and current clinical practice guidelines recommend collaborative treatment from a multidisciplinary team including, at least, a medical practitioner, a mental health professional and a dietitian [33, 34]

We are also moving towards routinely including families, other supports and peer workers in the treatment team. In particular, there is an increasing awareness of the need to consider the voice of consumers; one third of the ‘three-legged stool’ of research evidence, clinical expertise and lived experience [35]. Recovery from an ED is seen as a long and complex progression, less about a disease process as conceptualised by clinicians, and more about reclaiming a place in the world [36]. It is only by including the voice of lived experience in truly collaborative, co-designed research that we will better understand what people with EDs need at each stage of the recovery process [37]. Thankfully, carers and supports are now recognised as valued contributors to recovery and carers can receive training to participate fully in their loved one’s recovery [17, 38, 39].

#### Treatment modality

The results of treatment trials that have compared different treatment modalities over the past twenty years now allow us more choice in treatment delivery. For example, we can offer shorter treatments such as CBT-Ten [40], therapy via telehealth [41], Guided Self-Help [42–45], online interventions [46–51], multi-family interventions [52, 53] or Temperament Based Treatment with Supports (TBT-S) five-day treatment models [54], with confidence that similar outcomes to individual therapy can be achieved.

#### Timing of treatment

Because of what we now know about neuroplasticity, neuroprogression and critical windows for intervention, there has been a move to prioritise treatment for those with recent onset (*e.g.,* First Episode and Rapid Early Intervention service for Eating Disorders; FREED) [55]. Long waiting lists for specialist services have been managed by offering brief early interventions, e.g., Single Session Interventions [56, 57].

At the same time, we also have a responsibility to treat those who have had EDs for many years and to adapt our treatments to suit their priorities and needs, as we would do with all patients. Consumers tend to prefer the term “longstanding ED” to “severe and enduring ED,” or, ideally, no label regarding chronicity [58]. There are differences of opinion regarding EDs that have not responded to treatment, with some in the field [59] suggesting there are no features differentiating recent onset versus chronic EDs. While some people with longstanding EDs may appreciate a treatment approach that focuses on quality of life [60, 61], there is evidence that they can benefit from recovery-focused treatments that involve weight restoration, such as CBT-E [62].

### #3 Reconceptualizing recovery goals

#### Rethinking definitions of “recovery”

Broadening our definitions of ‘recovery’ may allow more flexibility in determining treatment strategies. Really listening to an individual’s expectations and goals (rather than imposing on them our research-based views of what constitutes “recovery”) [63–65] can help patients and therapists make informed, collaborative decisions about treatment delivery. Research that has drawn on those with lived experience of an ED tells us that recovery is much more than simply achieving a certain BMI, scoring below a particular score on an ED questionnaire and exhibiting few ED behaviours [66–68]. When we listen to those with lived experience, we learn that recovery is a complex, moving target that can be defined differently for different people [36, 37, 68–70].

#### Rethinking weight goals

Related to this, there has been a shift in our thinking around weight goals for people with AN. When it comes to proposing targets for weight restoration, we need to pay appropriate attention to each patient’s specific context and background, rather than prescribing a “normal” BMI. The FBT manual suggests a goal of a BMI at the 50th percentile on BMI-for-age charts, and the CBT-E manual suggests aiming for a BMI of 19–20. We strongly believe in considering premorbid weight (if known), childhood body type, family body type and weight history (highest and lowest weight) to guide us. One size does not fit all: a fifteen-year-old girl with a slight premorbid body frame and a lean family body type may not need to weight restore to the 50th percentile, while a similarly aged girl, who was in a larger body as a child and comes from a family with many high-weight members, may need to weight restore well beyond that level [71]. We must not let our own weight bias prevent us from advocating for sufficient weight restoration.

### #4: Building competency in working with diverse populations

The days of the ‘SWAG’ (skinny, white, affluent girls) stereotype in EDs are over. Our assessments and treatments must identify and address with sensitivity the needs of *all* individuals.

#### Neurodiversity

There is significant overlap between EDs and neurodivergent presentations such as autism [72–77] and attention deficit hyperactivity disorder [78–81]. Research has also demonstrated that feeding difficulties and eating disorders are overrepresented in people with intellectual disability [82, 83], giftedness [84–86], and Tourette’s Syndrome [87, 88]. While research into the links between EDs and neurodivergent presentations is just beginning to gather momentum (and the effects of starvation need to be distinguished from underlying neurodevelopmental conditions) the implications of this research has been signalled. For neurodivergent individuals, subtle differences in underlying approaches may be required regarding sensory, emotion and communication-based interventions [72, 89–91].

#### Gender diversity

It has become apparent that males, too, are affected by EDs—more commonly than we realised [92–94] and that current EBTs may not always provide a good fit for male patients [95–98]. Standardised measures of ED symptomatology, e.g., the Eating Disorders Examination [99] focusing on the drive for thinness, a common goal for females, do not capture the emerging concept of muscle dysmorphia [100–102].

The past decade has also seen a rise in the number of people identifying as transgender, non-binary or gender-fluid. Research has suggested that gay, bisexual and transgender individuals are at higher risk of developing an ED than their heterosexual and cisgender counterparts [103–108]. For transgender, non-binary and gender-fluid individuals, body image dissatisfaction occurs in the context of the person’s gender assigned at birth and current gender and sexual identity [103, 108, 109].

#### Racial diversity

Research has confirmed our clinical observation that EDs are found in all ethnicities, in similar rates to those of white populations. This includes indigenous populations in Australia [110]; and in the USA: African-Americans [111], Latinos [112] and Asian-Americans [113]; and in countries becoming westernised, e.g., Saudi Arabia [114]. Clinicians are becoming more open to identifying EDs in any ethnic group and providing culturally sensitive treatment.

#### Body diversity

Contrary to the common understanding in the 1990s and early 2000s, we now know that we cannot rely on Body Mass Index (BMI) as an indicator of health [115, 116]. Medical complications of caloric restriction and other ED behaviours can occur in individuals with bodies of all shapes and sizes, including individuals with high BMIs [7, 115–118]. Moreover, there is an increasing amount of robust medical evidence that challenges fundamental assumptions held by many (including medical professionals) about individuals in larger bodies, including the belief that “thinner is healthier” [115, 119]. Weight stigma [120, 121] has been shown to have adverse effects in relation to both social justice issues and medical implications [122–125]. Alongside this new information lies the often-ignored data clearly demonstrating that behavioural weight loss programs do not work in the long term and that dieting can set individuals up for binge eating and further weight gain [126–130]. Many clinicians have adopted the Health at Every Size® (HAES®) framework [131–133], a paradigm that has gathered momentum over the past decade, by helping to reduce ED behaviours and improve people’s relationship with food and movement, regardless of what occurs with their weight [7, 134].

Over the last quarter of a century there has been a rapid increase in the number of individuals opting for bariatric surgery which has resulted in another set of problems for individuals with EDs [135]. It is becoming more common to assess for and treat ED symptoms before any such surgery [134, 136–139].

### #5: Updating treatments to accommodate other sociocultural shifts

Changing societal views on diversity, as outlined above, reflect an attitudinal sociocultural shift. However, over the past two decades there have been other major sociocultural changes that can be responded to appropriately within EBT.

#### The rise of social media

Twenty years ago, there were no smart phones. Since then, the twenty-four hours per day access to images of unattainable or photoshopped bodies, plus the rise of ‘thinspiraton’, ‘fitspiration’ and ‘influencers’ has changed our culture in unimagined ways [140]. Particularly with young people, treatments can include psychoeducation about media literacy, which may encourage people to be critical of what they see in the media. Building resilience against the onslaught of social media and other sociocultural influences is crucial for bringing about enduring change [141–145].

#### Food insecurity

Having access to good nutrition is the foundation of ED treatment. The intersection of eating disorders and food insecurity (the limited or uncertain availability of nutritionally adequate and safe foods) has been highlighted by recent research [146–152] with 23% of young adults experiencing food insecurity [149]. The linear relationship between eating pathology and food insecurity cannot be ignored, with food insecurity being linked to increasing rates of BED [150], BN [151] and food restriction [152] in disadvantaged social groups.

### #6: Considering new ED diagnoses and advances in treatments for other conditions

#### Changes in the DSM

Twenty years ago, we were working from the Diagnostic and Statistical Manual of mental disorders, Fourth Edition, Text Revision [153]. In 2013, the Diagnostic and Statistical Manual of mental disorders, Fifth Edition [154] introduced two new ED diagnoses. The recognition of binge eating disorder (BED) has facilitated the development and testing of treatments for what is now regarded as the most common ED, both pharmacological [155] and psychological [156]. The introduction of ARFID has been followed by the development of specific interventions [157].

#### Incorporating specific modules into EBTs

Researchers and clinicians have started to broaden their scope in the quest to provide better care for people with EDs, applying treatments often developed and tested for other mental health conditions. CBT-E and FBT have been adapted by adding modules tailored to the individual’s needs and based on their specific formulation. Examples include modules focusing on: perfectionism [158–163]; strategies drawn from Dialectical Behaviour Therapy [164–167]; Cognitive Remediation Therapy (CRT) [89, 168, 169] or Cognitive Remediation and Emotion Skills Training (CREST) for rigid thinking [170–172]; TBT-S [17] for harnessing an individual’s traits for recovery; imagery rescripting [173–176]; or schema therapy [177, 178] to tackle core beliefs.

#### Incorporating physical activity into ED treatment

Neither of the two main EBTs focus on managing compulsive exercise. However, there is an awareness of the strong connection between driven movement and AN (mainly from animal studies) [179, 180] and we now have specific treatment approaches to address compulsive exercise [181–183]. There has also been a positive move toward integrating appropriate physical activity into treatment protocols [184–190].

### #7: Updating research design

As we embrace the idea of updating our current EBTs for EDs, we must also turn our hand to moving forward with innovative research. The following suggestions may yield new and relevant insights for all ED presentations.

#### Moving away from the traditional Randomised Controlled Trial (RCT) design

There has been extensive acknowledgement of the limitations of research to date in the ED field [191]. The late 1990s heralded an era dominated by RCTs comparing different treatments for EDs [192]. Such large clinical trials have become too expensive to conduct rigorously and, while RCTs have contributed enormously to the field, their findings have traditionally been disappointing, with differences in outcomes between the treatments rarely detected (the “Dodo Bird” effect) [193]. As a field we have begun to think creatively about applied research designs that are practical and targeted, and which provide answers to questions that are current and relevant [194]. These may include well-powered trials of brief, focused interventions including Single Session Interventions [195] and the careful reporting of case series or case studies [196, 197].

#### Adopting mixed-method research designs

It is possible to adopt different mixed-method research formats. For example, using qualitative as well as quantitative designs in a complementary way to add richness and colour to numbers and statistics [198, 199] or using pooled data sets or data from large registries which would have the added advantage of requiring different research teams to cooperate in order to find answers [200–206]. We are also listening to the voice of lived experience with regards to research. The ED field has been encouraged to engage in more meaningful co-design involving consumers, clinicians and researchers, with the potential to drive better outcomes [35, 194]. It will take courage to partner with those with lived experience and encourage them to lead the research process. It is only with truly collaborative, co-designed, research that we can redefine recovery [36, 37]. Co-design has many benefits, such as empowering consumers to seek out EBT via a co-designed checklist [194].

#### Taking advantage of new technologies

Future research will continue to be shaped by technological advances, particularly in the cutting-edge fields of genetics, neurobiology, neuromodulation, digital technology and psychopharmacology [19, 207]. Researchers can take advantage of new technologies to continue to progress research in the field of EDs.

## Summary

Clinicians specialising in the treatment of people with EDs must embrace new knowledge, new technologies, new treatment approaches and cultural shifts. Patients and families are asking for more individualised and personalised treatment. Many clinicians already report modifying EBTs [208]. However, we need to make sure that we adapt our robust EBTs thoughtfully and purposefully, so that we do not engage in unintentional “therapist drift” [209]. With such an approach—using and properly evaluating EBTs augmented by additional interventions—we can improve our treatments to help people move beyond their ED and flourish. A broadened, more flexible delivery of EBTs opens the door to innovative research designs that may start to bridge the often-cited gap between research and clinical practice.

## Data Availability

Not applicable.

## Abbreviations

ED: Eating disorders
CBT-BN: Cognitive Behavioural Bulimia Nervosa
CBT-E: Enhanced cognitive behaviour therapy
AN: Anorexia nervosa
FBT: Family based treatment
EBT: Evidence based treatment
MANTRA: Maudsley anorexia nervosa treatment for adults
SSCM: Specialist supportive clinical management
ARFID: Avoidant restrictive feeding and intake disorder
BMI: Body mass index
DSM: Diagnostic and statistical manual of mental disorders
BED: Binge eating disorder
CRT: Cognitive remediation therapy
EMDR: Eye movement desensitization and reprocessing
CREST: Cognitive remediation and emotional skills training
TBT-S: Temperament based treatment with supports
HAES: Health at every size
FREED: First episode and rapid early intervention service for eating disorders
RCT: Randomised controlled trial
